# Hippocampal T1WI radiomics- and clinical feature-based models for predicting early mild cognitive impairment in secondary hydrocephalus

**DOI:** 10.3389/fnagi.2025.1672254

**Published:** 2025-12-16

**Authors:** Xiaofeng Wang, Ziao Xu, Bohang Liu, Xuefei Ji, Liao Guan, Lei Ye, Hongwei Cheng

**Affiliations:** Department of Neurosurgery, First Affiliated Hospital of Anhui Medical University, Hefei, China

**Keywords:** mild cognitive impairment, radiomics, clinical feature, machine learning, secondary hydrocephalus

## Abstract

**Introduction:**

Mild cognitive impairment (MCI) represents the initial stage of dementia, and early diagnosis is crucial in clinical practice. This study aimed to investigate the predictive performance of three models based on clinical features, radiomics features of hippocampal T1-weighted imaging, and a combination of these features for identifying MCI in patients with secondary hydrocephalus.

**Methods:**

Of the 378 patients with secondary hydrocephalus, 124 were ultimately included in the study and divided into two cohorts: those with Mild Cognitive Impairment (MCI, *n* = 49) and those without MCI (*n* = 75). The samples were randomly stratified into a training set (34 MCI and 52 non-MCI patients) and a validation set (15 MCI and 23 non-MCI patients). Radiomic features from the bilateral hippocampi were extracted based on the region of interest, and the optimal parameters were selected through dimensionality reduction. Predictive models were constructed using clinical data, radiomic data, and a combination of both, with the radiomic score being utilized. The performance of each model was then assessed in both training and validation sets. Additionally, the diagnostic performance of the optimal model was compared with that of the Montreal Cognitive Assessment (MoCA) Scale.

**Results:**

In the clinical model, the disease course, serum uric acid, serum cystatin C, and the lateral ventricular temporal horn ratio emerged as independent risk factors for MCI following hydrocephalus. In the radiomics model, four optimal hippocampal features were identified. The AUC values for the clinical, radiomics, and combined models in the training/validation sets were 0.827 (0.736 ~ 0.919)/0.812 (0.666 ~ 0.957), 0.864 (0.790 ~ 0.937)/0.849 (0.724 ~ 0.974), and 0.937 (0.889 ~ 0.985)/0.907 (0.804 ~ 1.000), respectively. The combined model exhibited higher AUC values than the MoCA scale in both datasets. There was a significant difference in the training set, and while the validation set showed a consistent trend, it did not achieve statistical significance.

**Conclusion:**

The combined model achieved optimal performance and demonstrated superior predictive capabilities for MCI in the patients with secondary hydrocephalus outperforming other models.

## 1 Introduction

Hydrocephalus is a common neurological disorder, that leads to a variety of symptoms, including headaches, vomiting, vision problems, and cognitive impairment. An epidemiological study reported that the incidence of mild cognitive impairment (MCI) is as high as 78% among patients with idiopathic normal pressure hydrocephalus (iNPH) (Cai et al., 2025). In contrast, there is a lack of relevant data on the proportion of patients with secondary hydrocephalus who develop cognitive impairment. MCI is considered an early phase of dementia (Anderson, 2020; Petersen, 2016). In iNPH, cognitive impairment may be reversible, especially with early diagnosis and surgical treatment. If not properly treated, patients typically progress from MCI to dementia (Livingston et al., 2020). Therefore, evaluating whether patients with hydrocephalus have MCI in the early stage is important for formulating a personalized treatment plan.

Some studies have indicated that the periventricular white matter of patients with iNPH often shows high signals (He et al., 2025; Liu et al., 2025). This phenomenon is caused by interstitial edema and axonal stretching resulting from abnormal cerebrospinal fluid dynamics. Diffusion tensor imaging (DTI) can non-invasively assess the integrity and directionality of white matter fiber tracts. Parameters such as the reduction in fractional anisotropy (FA) and the increase in mean diffusivity (MD) suggest damage to the white matter structure (Parker et al., 2025). Vipin et al. (2018) found that in MCI patients, there are changes such as FA reduction and MD increase in regions such as the internal capsule and corpus callosum, indicating that the preservation of brain white matter is negatively correlated with cognitive impairment. In iNPH, the hippocampus may be mechanically compressed due to the expansion of the ventricles, resulting in its volume reduction or structural deformation. More importantly, DTI data show that even if the macroscopic volume remains unchanged, the microscopic structure of the hippocampus has undergone significant damage, which is closely related to memory dysfunction (Lilja-Lund et al., 2020). In neuropathology, the core of the neuropathological basis of iNPH cognitive impairment lies in the chain reaction caused by ventricular expansion, mainly involving mechanical axonal injury (Fallmar et al., 2021), which in turn affects the hippocampus and its fiber connections (Wang et al., 2020). More importantly, the reduction in cerebral blood flow in the hippocampal region triggered by this leads to chronic ischemia and hypoxia, and the hippocampal neurons are extremely sensitive to hypoxia, which will disrupt their energy metabolism and ultimately lead to neuronal damage and cognitive decline (Ji et al., 2021; Johanson et al., 2008). Although the initial causes of secondary hydrocephalus vary, the resulting ventricular dilation and the physical effects on the brain tissue are similar.

Radiomics non-invasively captures intrinsic heterogeneity by mining neuroimaging data and applying artificial intelligence, machine learning, or statistical methods to analyze high-dimensional datasets, thereby obtaining radiomic features that cannot be identified by visual observation (Scapicchio et al., 2021). This technology has been applied in the diagnosis of idiopathic normal pressure hydrocephalus (iNPH) (Lee et al., 2025) and the prediction and classification of hydrocephalus after intracerebral hemorrhage (Zhu et al., 2025). MCI is reportedly associated with hippocampal damage (Lin et al., 2025; Sung et al., 2024; Yang C. et al., 2024). Numerous studies have demonstrated that machine learning or deep learning models developed based on hippocampal radiomics can effectively achieve the diagnosis and classification of MCI. Wang et al. (2022) found that the radiomics model based on the structural images of the hippocampus performed better in the diagnosis of Alzheimer’s disease (AD) than the model using textural features of the amplitude of low frequency fluctuation (ALFF), while the model based on ALFF had better discrimination ability in the diagnosis of mild cognitive impairment than the model including structural images. Additionally, Yin et al. (2024) demonstrated that the random forest model of the T1-weighted imaging-based hippocampal radiomics played a superior role in identifying AD-related MCI and dementia. However, whether hippocampal radiomics can predict early MCI in patients with secondary hydrocephalus remains unknown. In this study, we construct relevant models based on hippocampal T1-weighted imaging (T1WI) radiomics and clinical features to investigate their value in predicting MCI in patients with secondary hydrocephalus.

## 2 Materials and methods

### 2.1 Patient selection

We retrospectively evaluated 378 patients with hydrocephalus admitted to the First Affiliated Hospital of Anhui Medical University from January 2021 to December 2024. All patients were adults diagnosed with hydrocephalus based on an expert consensus in China. We used the mini-mental state examination (MMSE) scale to exclude dementia. The time between the onset of hydrocephalus and the primary disease was greater than 2 weeks, and the patients did not receive pharmacological or surgical treatment before admission. We assessed the patients’ magnetic resonance imaging (MRI) results. Their demographic and clinicopathological data were also recorded. The exclusion criteria included: (1) an acute hydrocephalus duration of less than 2 weeks; (2) patients with an MMSE score less than 24; (3) patients with altered consciousness; (4) primary injury in cognition-related cerebral areas; (5) patients with other neurodegenerative or neuropsychiatric disorders, such as AD, Parkinson’s disease (PD), frontotemporal dementia (FTD), dementia with Lewy bodies (DLB), and ischemic cerebrovascular disease; (6) patients with other diseases that might influence cognition, such as hypothyroidism and vitamin deficiencies and toxicities; (7) patients with systemic inflammatory diseases or malignant tumors; (8) patients with incomplete clinical data; and (9) patients whose MRI had artifacts or poor quality that affected preprocessing or region of interest (ROI) delineation. Finally, 124 patients with hydrocephalus were enrolled in the study, including 71 males and 53 females.

Evaluation for MCI: All patients underwent a comprehensive neuropsychological assessment to determine their status of MCI. The diagnosis was strictly based on the 2003 International Working Group diagnostic criteria for MCI (Winblad et al., 2004) and the criteria outlined in the Chinese guidelines for diagnosing and treating MCI (Writing Goup of the Dementia and Cognitive Society of Neurology Committee of Chinese Medical Association, Alzheimer’s Disease Chinese, 2010). The core diagnostic criteria included: (1) subjective cognitive decline reported by the patient or an informant; (2) objective evidence of impairment in one or more cognitive domains, defined as a performance falling more than 1.5 standard deviations below the age- and education-adjusted norms; (3) preserved basic activities of daily living; and (4) failure to meet the criteria for dementia.

Cognitive assessment was conduct using a standardized battery of neuropsychological tests, which covered multiple core cognitive domains: (1) Global cognition was evaluated using the Montreal Cognitive Assessment (MoCA) and the Mini-Mental State Examination (MMSE). In the clinical diagnostic workflow, the MMSE served as an initial screening tool, primarily used to exclude patients with severe dementia (typically defined by an MMSE score <24), given its recognized lower sensitivity for detecting MCI. In contrast, the MoCA, known for its higher sensitivity in identifying MCI, was employed as the primary tool to differentiate between MCI and cognitively normal individuals. (2) Memory domain was evaluated using the Auditory Verbal Learning Test-Delayed Recall and the Rey-Osterrieth Complex Figure Test-Delayed Recall. (3) Executive function/attention was assessed using the Digit Span Test and the Trail Making Test Parts A & B. (4) Language Function: Evaluated using the Boston Naming Test and the Verbal Fluency Test. (5) Visuospatial function was assessed using the Rey-Osterrieth Complex Figure Test-Copy.

To account for the influence of educational level, MoCA scores were adjusted (by adding 1 point for patients with ≤12 years of education). An adjusted MoCA score of <26 was used as an auxiliary diagnostic cut-off for MCI. All test scores were corrected for age and education based on established Chinese normative data.

To maximize diagnostic objectivity and grouping accuracy, the following quality control measures were implemented: (1) Blinding Procedures: The neuropsychological assessors were blinded to the patients’ imaging data. Conversely, the radiomics analysts responsible for hippocampal segmentation and feature extraction were blinded to the patients’ final clinical diagnoses and detailed neuropsychological results. (2) Expert Review: All preliminary MCI diagnoses and cases with diagnostic uncertainty underwent a secondary review by an independent panel led by a senior neurologist. This panel made the final determination on MCI grouping after a comprehensive review of all available clinical histories, neuropsychological reports, and conventional imaging findings.

**Figure 1:**
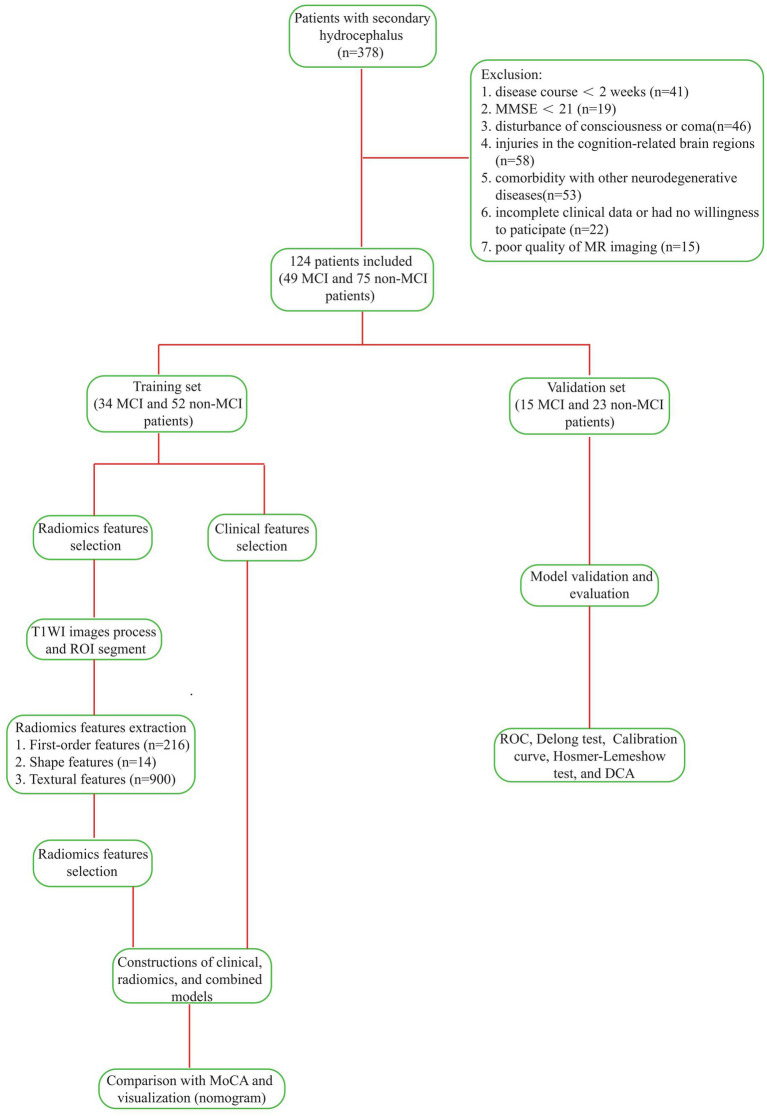
Technology Roadmap of the experiment.

Finally, we categorized the enrolled patients into two groups: 49 with MCI and 75 without MCI. Utilizing R4.4.0 software,^1^ we randomly assigned patients into a training set (*n* = 86, comprising 34 MCI cases and 52 non-MCI cases) and a validation set (*n* = 38, with 15 MCI cases and 23 non-MCI cases) at a ratio of 7:3, setting the random seed to 3. The proportional distribution of categories was consistently maintained across both sets, as depicted in Figure 1 (Technology Roadmap). The Ethics Committee of the First Affiliated Hospital of Anhui Medical University approved this study. All patients provided written informed consent.

### 2.2 MRI protocol

To perform axial T1WI scans of the cranial brain, we utilized a 3.0 T MR scanner (Prisma, Siemens, Erlangen, Germany), employing the following parameters: TR = 2000 ms, TE = 7.4 ms, flip angle = 150°, slice thickness = 6 mm, inter-slice gap = 1.8 mm, and voxel size = 0.6875 × 0.6875 × 6 mm^3^.

### 2.3 Clinical data collection

Relevant data for all participants were obtained by consulting the electronic medical record system and conducting follow-up interviews. The collected data encompassed sex, age, BMI, education level, etiology, disease course, smoking status, alcohol consumption, hypertension, diabetes, hyperlipidemia, history of craniocerebral surgery, initial lumbar puncture pressure (ILPP), and laboratory indicators, such as hemoglobin (Hb), albumin (ALB), lactate dehydrogenase (LDH), creatinine (Cr), retinol-binding protein (RBP), soluble uric acid (sUA), soluble cystatin C (sCysC), sodium, total protein in cerebrospinal fluid (CSF-TP), chlorine in CSF (CSF-Cl), and glucose in CSF (CSF-Glu). Additionally, a cerebral T1WI MRI scan was performed prior to lumbar puncture or surgery. The conventional MRI imaging features assessed included: (1) the medial temporal lobe atrophy rating (MTA) scale; (2) the lateral ventricular temporal horn ratio (LVTH; the ratio between the shortest and longest distances between the two temporal angles of the lateral ventricles on the same plane); and (3) the transverse-to-anteroposterior diameter ratio of the third ventricle (TVR). The transverse diameter was measured as the maximum transverse distance between the lateral walls of the third ventricle at the anterior commissure-posterior commissure (AC-PC) plane on the axial images. The anteroposterior diameter was measured as the maximum distance from the anterior commissure to the posterior commissure or the aqueductal opening on the sagittal or axial images. It is important to note that in this study, “disease course” was defined as the duration from the initial clinical diagnosis of the primary disease causing hydrocephalus to the first diagnosis of hydrocephalus confirmed by imaging and clinical criteria.

### 2.4 Image preprocessing

T1WI sequences from MRI were exported in DICOM format from the Picture Archiving and Communications System (PACS). Initially, we utilized the “pydicom” package in Python (version 3.7.0)^2^ to read the DICOM data, followed by conversion to nii.gz format using the “nibabel” package. Subsequently, we applied the “N4BiasFieldCorrection” function from the “ANTsPy” package in Python to perform N4 bias field correction on the MRI images. Finally, we used normalization function of the “nibabel” package in Python to standardize and normalize MRI image intensity values, ensuring data consistency across different images.

### 2.5 Hippocampal segmentation

Extensive research has shown the considerable diagnostic value of hippocampal radiomic features in MCI. Building on this evidence, the current study conducted manual delineation of the bilateral hippocampal regions on T1WI sequences and extracted their radiomic features for further analysis.

**Figure 2:**
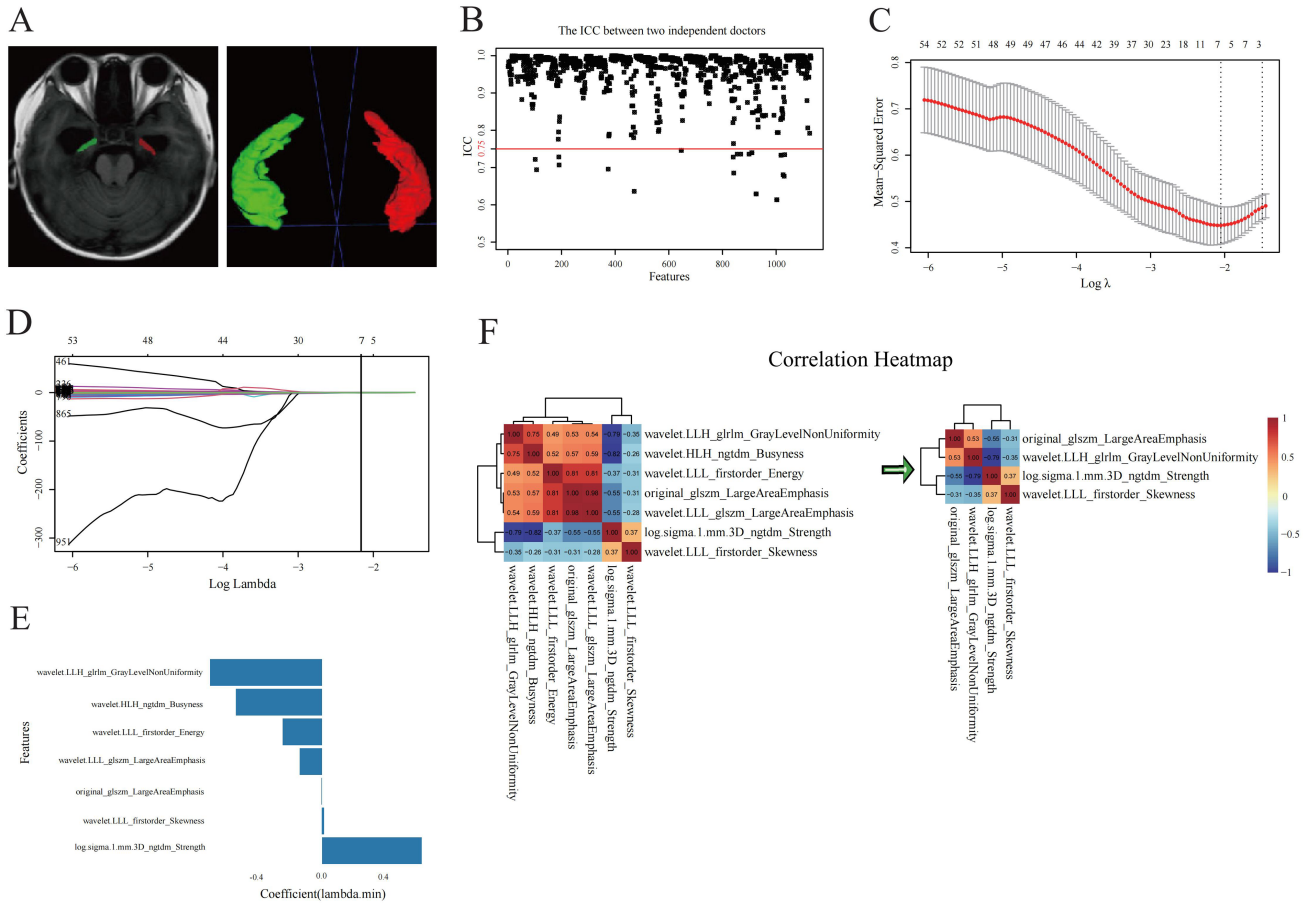
Selection of optimal radiomics features. **(A)** Hippocampal segmentation of three-dimensional volumes of interest of hippocampi. **(B)** ICC test and **(C–E)** LASSO regression analysis for radiomic features of hippocampi. **(F)** correlation analysis for the evaluation of redundancy among radiomic features.

The normalized T1WI sequences from the MRI were imported into the open-source software ITK-SNAP (Version 4.0).^3^ There, manual slice-by-layer segmentation of the ROI, the bilateral hippocampi, was executed on axial images. The software automatically produced complete three-dimensional volumes of interest (VOIs) for the hippocampi, as depicted in Figure 2A (VOI delineation diagram).

We subsequently performed analogous bilateral hippocampal delineation on T1WI images of 40 randomly selected patients. For the segmentation of the hippocampus, a neurosurgeon completed the bilateral hippocampal segmentation for all patients, while a senior radiologist independently selected T1WI images of 40 patients to conduct analogous bilateral hippocampal delineation. Both medical professionals were blinded to the clinical information and outcomes of these patients. ROI delineation was performed in accordance with the Radiation Therapy Oncology Group (RTOG) hippocampal contouring consensus guidelines.^4^ The definition of boundaries was as follows: anterior boundary: posterior border of the amygdala; posterior boundary: the junction between the crus of the fornix and the quadrigeminal cistern; upper boundary: the top of the temporal horn of the lateral ventricle; lower boundary: the hippocampal sulcus; medial boundary: the ambient cistern and lateral margin of the mesencephalon; and lateral boundary: the medial wall of the temporal horn of the lateral ventricle.

### 2.6 Radiomic features extraction and selection

Before feature extraction, all hippocampal region of interest (ROI) images were resampled to a standardized in-plane resolution of 0.69 × 0.69 mm^2^, while preserving the original slice thickness of 6 mm. This resulted in a uniform voxel size of 0.69 × 0.69 × 6 mm^3^. Trilinear interpolation was applied to the image intensity values, and nearest-neighbor interpolation was used for the corresponding masks to prevent interpolation-induced alterations in ROI labels. Radiomic features of VOIs were extracted using the open-source “Pyradiomics” toolkit^5^ (Cha et al., 2014) in Python, adhering strictly to the Imaging Biomarker Standardization Initiative (IBSI) guidelines (Zwanenburg et al., 2020). In the step of gray-level discretization (also known as intensity binning), we employed a fixed bin width method with a bin width value of 25. The two groups of radiomic features extracted from the VOIs of the 40 randomly selected patients were analyzed using the interclass correlation coefficients (ICC) test. We retained stable features with ICC values greater than 0.75. The radiomic feature values were standardized using the Z-score normalization method. Z-score normalization parameters were calculated solely from the training set and subsequently applied to both the training and validation sets. In the training set, we performed dimensionality reduction and selection of radiomic features using statistical analyses. The optimal radiomic features, which were strongly associated with MCI were selected to construct a logistic regression model. The optimal radiomic features selected were multiplied by the corresponding weighting coefficients, summed up, and added to the intercept to obtain the radiomics score (Rad-score). The Rad-score is equivalent to the predictive probability of MCI positivity in each patient with hydrocephalus. The Rad-score was calculated using the formula: Rad-score = β0 + *Σ* (βi × Xi), where β0 represents the intercept, βi denotes the coefficient, and Xi corresponds to the value of the selected features.

### 2.7 Model construction and validation

In the training set, after identifying the clinically independent predictors of MCI in patients with hydrocephalus, a logistic regression-based clinical predictive model was constructed. The optimal neuroimaging features selected were also utilized to construct a radiomics-related model using logistic regression algorithms, and the Rad-score was calculated accordingly. We then integrated the Rad-score of radiomic and clinical features to build a combined model. A nomogram was created to visualize the predictive outcomes of the combined model. Subsequently, the predictive performance of each model was validated in the validation set. Calibration curves were employed to evaluate the accuracy of the models and the consistency between the predicted probabilities and the observed outcomes. A decision curve analysis (DCA) was used to assess the clinical application value of each model. The diagnostic performance of the optimal model was compared with that of the MoCA.

### 2.8 Resampling validation

To evaluate the robustness of model performance estimates and minimize the random bias introduced by a single data split, we concurrently implemented two validation strategies within the training set: 5-fold cross-validation and 500-repetition Bootstrap resampling validation with 500 iterations.

5-fold cross-validation: The training set was randomly partitioned into five mutually exclusive subsets of approximately equal size. Each subset was sequentially used as the validation set, while the remaining four subsets were combined to form the training set for model development and validation. This process was repeated five times, ensuring that each subset was used for validation exactly once. The performance metrics [e.g., Area under the Curve (AUC), accuracy] from all five folds were aggregated, and their mean and standard deviation were calculated to provide a comprehensive assessment of the model’s average performance and stability. Bootstrap resampling validation with 500 iterations: We conducted 500 bootstrap replicates by randomly drawing samples with replacement from the original training set, each time generating a bootstrap sample of the same size as the original training set. For each bootstrap sample, a model was trained, and its performance was assessed both on the bootstrap sample itself and on the corresponding out-of-bag (OOB) samples (i.e., the portion of the original training set not included in the bootstrap sample). The optimism statistic was calculated by determining the mean difference between the performance metrics (primarily AUC) on the bootstrap samples and the OOB samples across all 500 replicates. Finally, the bootstrap-corrected performance estimate, which offers a less biased assessment of the model’s generalization ability, was derived by subtracting this optimism statistic from the apparent performance observed when the model was trained on the complete original training set.

The aforementioned internal validation procedures were uniformly applied to the clinical, radiomics, and integrated models developed in this study.

### 2.9 Temporal external validation

To rigorously evaluate the generalizability and clinical applicability of the developed models, an additional temporal external validation was conducted. The validation cohort comprised a completely new and independent prospective patient cohort (*n* = 33). These patients were enrolled consecutively at the same medical center, following the identical inclusion and exclusion criteria, after the recruitment period for the initial development cohort had ended (from January 1, 2025, to October 20, 2025). This dataset was entirely independent of any model development or prior testing processes, Thusconstituting a pristine, standalone validation set for the objective assessment of the models’ temporal generalizability.

### 2.10 Statistical methods

SPSS (version 26.0, IBM) and R software (Version 4.4.0, see text footnote 1, respectively) were utilized for statistical analysis. The normality of the quantitative data was assessed using the Kolmogorov–Smirnov test. Normally distributed quantitative data were expressed as the mean ± standard deviation (x ± s), and inter-group comparisons were conducted using the Student *t*-test. In cases where quantitative data did not follow a normal distribution, they were expressed as the quartiles and compared using the Mann–Whitney U test. Categorical data were expressed as frequencies [percentages (%)] and analyzed using chi-square or Fisher’s exact tests. Clinical or radiomic variables were screened using univariate, multivariate logistic regression, Lasso regression, and Spearman’s correlation coefficient analyses for dimensionality reduction. The combined models were constructed using logistic regression equations. Inter-group comparisons of Rad-scores were performed using the Wilcoxon test. The predictive value of each logistic regression model for the occurrence of MCI in hydrocephalus was evaluated using the area under the curve (AUC) of the receiver operator characteristic (ROC) curve. Differences in AUC were compared using DeLong’s test, and a *p* < 0.05 was considered statistically significant. The calibration of all models was evaluated using calibration curves. The goodness-of-fit was analyzed using the Hosmer–Lemeshow test, and a *p* > 0.05 indicated that the model fits well. The clinical applicability of each model was evaluated using a DCA.

## 3 Results

### 3.1 Baseline data

All participants were randomly assigned to training and validation sets at a ratio of 7:3. We compared the demographic and clinicopathological data between the two sets, and the results indicated no statistical difference, suggesting that the allocation method between the training and validation sets was reasonable (Table 1).

**Table 1:** Demographic and clinicopathological features of training and validation sets.

Variables	Total (*n* = 124)	Validation set (*n* = 38)	Train set (*n* = 86)	*p*
Gender, *n* (%)				0.919
Female	53 (42.74%)	17 (44.74%)	36 (41.86%)	
Male	71 (57.26%)	21 (55.26%)	50 (58.14%)	
Age(years), *n* (%)				0.850
18 ~ 29	24 (19.35%)	8 (21.05%)	16 (18.60%)	
30 ~ 59	70 (56.45%)	20 (52.63%)	50 (58.14%)	
≥60	30 (24.19%)	10 (26.32%)	20 (23.26%)	
BMI(kg/m^2^), *n* (%)				0.761
<18	9 (7.26%)	3 (7.89%)	6 (6.98%)	
18 ~ 23	56 (45.16%)	15 (39.47%)	41 (47.67%)	
24 ~ 27	49 (39.52%)	16 (42.11%)	33 (38.37%)	
≥28	10 (8.06%)	4 (10.53%)	6 (6.98%)	
Education, *n* (%)				0.981
Illiteracy	24 (19.35%)	7 (18.42%)	17 (19.77%)	
Primary school	34 (27.42%)	10 (26.32%)	24 (27.91%)	
Middle school	36 (29.03%)	12 (31.58%)	24 (27.91%)	
University	30 (24.19%)	9 (23.68%)	21 (24.42%)	
Disease course (months), *n* (%)				0.498
<3	30 (24.19%)	7 (18.42%)	23 (26.74%)	
3 ~ 6	37 (29.84%)	10 (26.32%)	27 (31.40%)	
6 ~ 12	30 (24.19%)	10 (26.32%)	20 (23.26%)	
>12	27 (21.77%)	11 (28.95%)	16 (18.60%)	
Smoking, *n* (%)				0.165
No	82 (66.13%)	29 (76.32%)	53 (61.63%)	
Yes	42 (33.87%)	9 (23.68%)	33 (38.37%)	
Alcohol consumption, n (%)				0.365
No	86 (69.35%)	29 (76.32%)	57 (66.28%)	
Yes	38 (30.65%)	9 (23.68%)	29 (33.72%)	
Primary neurological disorders, n(%)				1.000
Traumatism	13 (10.48%)	4 (10.53%)	9 (10.47%)	
Hemorrhage	22 (17.74%)	7 (18.42%)	15 (17.44%)	
aSAH	32 (25.81%)	10 (26.32%)	22 (25.58%)	
Infarction	7 (5.65%)	2 (5.26%)	5 (5.81%)	
Tumor	19 (15.32%)	6 (15.79%)	13 (15.12%)	
Infection	14 (11.29%)	4 (10.53%)	10 (11.63%)	
Other	17 (13.71%)	5 (13.16%)	12 (13.95%)	
Hypertension, n (%)				0.847
No	75 (60.48%)	22 (57.89%)	53 (61.63%)	
Yes	49 (39.52%)	16 (42.11%)	33 (38.37%)	
Diabetes, *n* (%)				0.482
No	98 (79.03%)	32 (84.21%)	66 (76.74%)	
Yes	26 (20.97%)	6 (15.79%)	20 (23.26%)	
Hyperlipemia, *n* (%)				0.443
No	89 (71.77%)	25 (65.79%)	64 (74.42%)	
Yes	35 (28.23%)	13 (34.21%)	22 (25.58%)	
Neurosurgical history, *n* (%)				1.000
No	81 (65.32%)	25 (65.79%)	56 (65.12%)	
Yes	43 (34.68%)	13 (34.21%)	30 (34.88%)	
Hemoglobin(g/L), mean ± SD	131.06 ± 16.47	130.08 ± 16.61	131.49 ± 16.49	0.664
Albumin(g/L), mean ± SD	41.04 ± 4.26	41.57 ± 4.07	40.80 ± 4.34	0.345
Lactate dehydrogenase(U/L), M[Q1, Q3]	171.00 [151.00,206.66]	191.00 [155.50,205.75]	165.00 [151.00, 206.89]	0.207
Creatinine(μmol/L), mean ± SD	60.56 ± 19.51	61.80 ± 18.20	60.01 ± 20.14	0.627
Serum UA(μmol/L), mean ± SD	316.15 ± 82.41	319.26 ± 85.35	314.78 ± 81.55	0.785
Serum Cys-C(mg/L), M[Q1, Q3]	1 0.00 [0.86, 1.16]	1.04 [0.91, 1.22]	0.98 [0.86, 1.15]	0.182
RBP(mg/L), mean ± SD	45.60 ± 11.63	46.04 ± 16.08	45.41 ± 9.12	0.824
Na^+^(mmol/L), M[Q1, Q3]	139.80 [138.57, 141.72]	140.50 [139.22, 142.25]	139.30 [138.43, 141.48]	0.063
FLPP(mmH_2_O), M[Q1, Q3]	180.00 [150.00, 220.00]	192.50 [140.00, 247.50]	180.00 [156.25, 210.00]	0.411
CSF total protein(g/L), M[Q1, Q3]	0.72 [0.47, 1.09]	0.85 [0.48, 1.06]	0.66 [0.46, 1.13]	0.468
CSF Chlorine(mmol/L), M[Q1, Q3]	123.00 [121.20, 125.15]	124.26 [121.97, 126.00]	122.82 [121.20, 124.90]	0.127
CSF Glucose(mmol/L), M[Q1, Q3]	3.63 [3.38, 4.10]	3.63 [3.39, 4.58]	3.63 [3.39, 4.06]	0.788
MTA scale, n (%)				0.938
Grade 0	8 (6.45%)	3 (7.89%)	5 (5.81%)	
Grade 1	14 (11.29%)	5 (13.16%)	9 (10.47%)	
Grade 2	57 (45.97%)	18 (47.37%)	39 (45.35%)	
Grade 3	23 (18.55%)	6 (15.79%)	17 (19.77%)	
Grade 4	22 (17.74%)	6 (15.79%)	16 (18.60%)	
TVR, mean ± SD	0.62 ± 0.19	0.63 ± 0.20	0.62 ± 0.18	0.834
Ratio of LVTH, mean ± SD	0.51 ± 0.12	0.50 ± 0.09	0.52 ± 0.13	0.346

### 3.2 Radiomic features selection

A total of 1,130 radiomic features were extracted, comprising 216 first-order statistical features, 14 morphological features, and 900 textural features. These texture features included 288 gray level co-occurrence matrixes (GLCM), 192 gray level run length matrices (GLRLM), 192 gray level size zone matrices (GLSZM), 60 neighborhood gray-tone difference matrices (NGTDM), and 168 gray level dependence matrices (GLDM).

**Figure 3:**
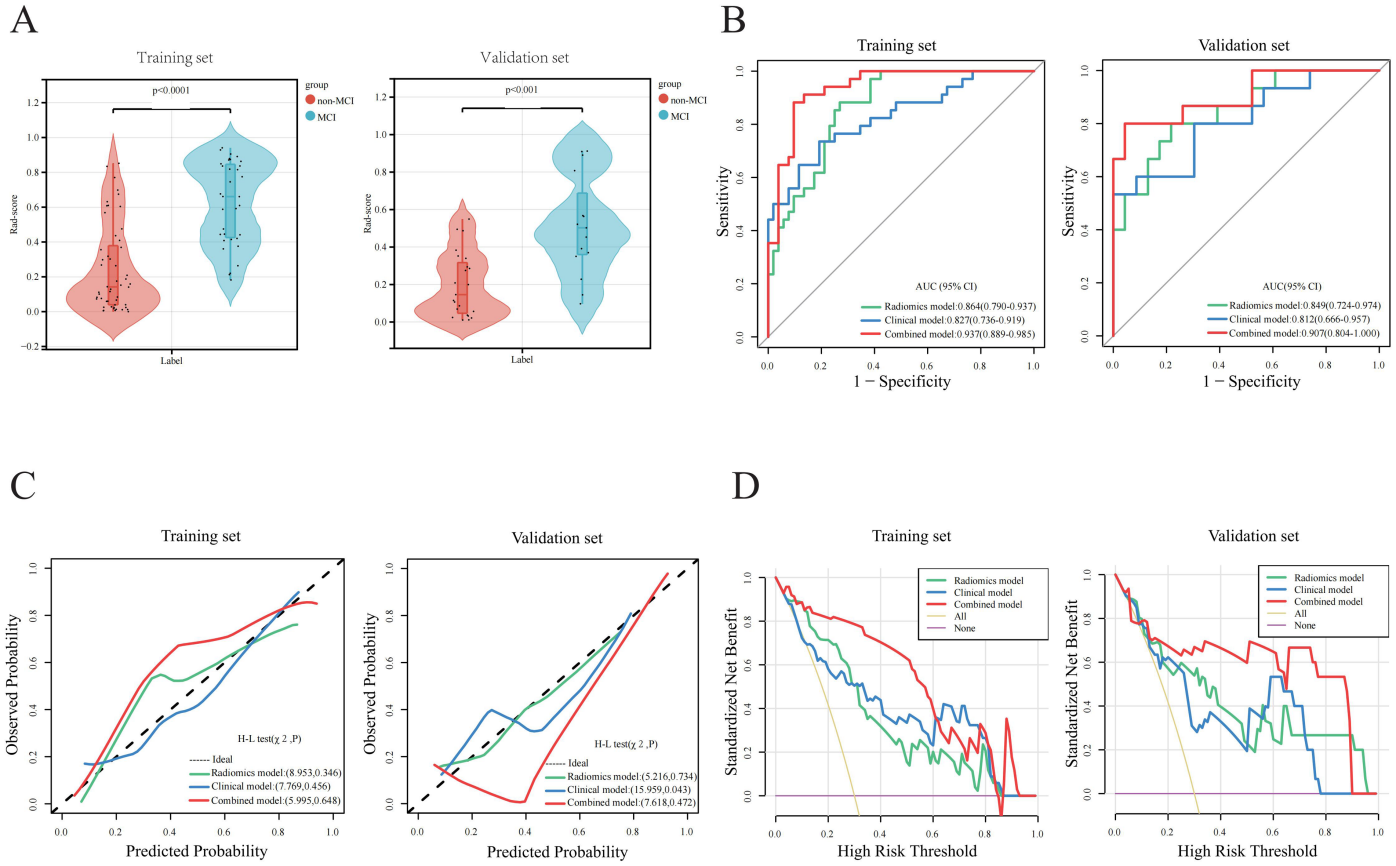
Parameter comparisons of model construction between training and validations set. **(A)** Rad-score comparisons. **(B)** AUC analysis comparisons. **(C)** Calibration curve comparisons. **(D)** Decision Curve Analysis (DCA).

We conducted an ICC test on two sets of radiomic features extracted from hippocampal VOIs of 40 identical patients. The results indicated that 1,102 radiomic features had ICC values ≥ 0.75 (Figure 2B). This method, allowed us to effectively evaluate the consistency of ROI delineations among physicians, thereby enhancing the reliability and stability of the radiomics analysis. Prior to screening, we normalized the radiomic feature values using the Z-score standardization method. A univariate analysis was conducted on each candidate independent variable in the training set, resulting in 1074 radiomics features remaining significant. Subsequently, we performed a LASSO regression analysis to reduce the dimensionality. Through 10-fold cross-validation, the optimal lambda (*λ*) value was calculated to minimize the binomial deviance of the model. Seven more significantly stable features with non-zero coefficients were selected: original-glszm-LargeAreaEmphasis, wavelet. LLH-glrlm-GrayLevelNonUniformity, log.sigma.1.mm.3D-ngtdm-Strength, wavelet. HLH-ngtdm-Busyness, wavelet. LLL-firstorder-Energy, wavelet. LLL-firstorder-Skewness, and wavelet. LLL-glszm-LargeAreaEmphasis, as shown in Figures 2C–E. We evaluated redundancy among the feature parameters in the correlation analysis of these seven features. A feature was removed if any pair of features had an absolute correlation coefficient exceeding 0.8, and this process was iteratively repeated until all pairwise correlations between features fell below 0.8. Ultimately, four optimal radiomic features were retained (Figure 2F). Therefore, collinearity among features was reduced, and the prediction performance and generalization ability of the model were improved. Finally, the four optimal radiomic features were multiplied by the corresponding weighting coefficients, summed up, and added to the intercept to calculate the Rad-score. In both the training and validation sets, the Rad-score of the MCI-positive group was significantly higher than that of the MCI-negative group, with statistically significant differences (Z = 241 and 52, respectively; Figure 3A).

### 3.3 Construction of radiomics and clinical models

In the training set, a multivariate logistic regression analysis was conducted based on the Rad-score to construct a radiomics model, and the predictive performance of the model was evaluated in the validation set. The AUC of the radiomics model in the training and validation sets were 0.864(0.790 ~ 0.937) and 0.849(0.724 ~ 0.974), respectively, indicating that the model’s classification performance was similar in both datasets (Figure 3B). Each calibration curve was close to the diagonal in both sets. The model’s Brier score in the training and validation sets was 0.157/0.164, the calibration slope was 1.000/1.290, and the calibration intercept was 0.000/0.742, respectively, (Supplementary Table 2). All these indicated that the model’s predicted probability matched the actual occurrence rate (Figure 3C). The result of the DCA demonstrated that in clinical application, the radiomics predictive model has favorable net benefits, with threshold probabilities ranging from 0.1 to 0.9 and 0.1 to 0.97 in the training and validation sets, respectively (Figure 3D). These results indicate that the radiomics model exhibited robust performance and strong generalizability.

Based on the radiomics grouping, we conducted a univariate analysis of each candidate clinical variable in the training set. The results revealed nine clinical features with statistical significance: disease course, MTA scale, sUA, sCys-C, RBP, ILPP, CSF-TP, TVR, and LVTH (Table 2). In the multivariate regression analysis, the disease course, sUA, sCysC, and LVTH remained statistically significant (Table 2). The AUC of the clinical model in the training and validation sets were 0.827 (0.736 ~ 0.919) and 0.812 (0.666 ~ 0.957), respectively (Figure 3B). Calibration curves for both groups indicated that the clinical model has excellent calibration accuracy without systematic bias (Figure 3C). The model’s Brier score in the training and validation sets were 0.160/0.180, the calibration slope were 1.000/0.854, and the calibration intercept were 0.000/−0.657, respectively, (Supplementary Table 2). The DCA demonstrated that the clinical predictive model has good net benefits, with threshold probabilities ranging from 0.20 to 0.95 in the training set and from 0.25 to 0.85 in the validation set, respectively (Figure 3D).

**Table 2:** Univariate and multivariate logistic regression analysis of clinical features.

Variables	*p* value	OR	B	SE	*p* value	OR	95% CI
Gender							
Male	0.732	0.858					
Age(years)							
30 ~ 60	0.626	1.348					
>60	0.260	2.200					
BMI(kg/m^2^)							
18 ~ 24	0.145	0.259					
24 ~ 28	0.230	0.325					
>28	0.560	0.500					
Education							
Primary school	0.853	0.889					
Middle school	0.129	0.366					
University	0.131	0.356					
Disease course (months)							
3 ~ 6	0.197	0.426	−1.013	1.049	0.334	0.363	0.047 ~ 2.838
6 ~ 12	0.186	2.292	2.283	1.140	0.045	9.804	1.049 ~ 91.615
>12	0.092	3.125	1.744	1.247	0.162	5.720	0.497 ~ 65.861
Smoking							
Yes	0.666	1.216					
Alcohol consumption							
Yes	0.495	0.724					
Primary neurological disorders							
Hemorrhage	0.461	1.875					
aSAH	0.676	0.714					
Infarction	0.872	0.833					
Tumor	0.514	0.556					
Infection	0.517	0.536					
Other	0.605	0.625					
Hypertension							
Yes	0.355	0.652					
Diabetes							
Yes	0.961	1.026					
Hyperlipemia							
Yes	0.393	0.640					
Neurosurgical history							
Yes	0.149	1.947					
MTA scale							
MTA scale1	0.923	1.143	−1.141	1.820	0.531	0.320	0.009 ~ 11.314
MTA scale2	0.911	0.875	−2.675	1.765	0.130	0.069	0.002 ~ 2.190
MTA scale3	0.068	9.600	0.234	1.711	0.891	1.264	0.044 ~ 36.191
MTA scale4	0.048	12.000	1.018	1.638	0.534	2.769	0.112 ~ 68.657
Hemoglobulin (g/L)	0.741	0.996					
Albumin(g/L)	0.178	0.931					
Lactate dehydrogenase (U/L)	0.173	0.993					
Creatinine (μmol/L)	0.532	0.993					
Serum Uric acid (μmol/L)	0.092	0.995	−0.011	0.005	0.039	0.989	0.978 ~ 0.999
Serum Cys-C (mg/L)	0.008	22.557	5.444	2.632	0.039	231.348	1.329 ~ 40277.280
RBP(mg/L)	0.092	1.043	0.045	0.043	0.295	1.046	0.962 ~ 1.137
Na^+^(mmol/L)	0.790	1.021					
FLPP(mmH_2_O)	0.083	1.010	0.016	0.013	0.209	1.017	0.991 ~ 1.043
CSF total protein(g/L)	0.024	3.613	1.481	0.856	0.084	4.398	0.821 ~ 23.557
CSF Chlorine (mmol/L)	0.446	0.953					
CSF glucose (mmol/L)	0.510	1.180					
TVR	0.032	16.510	4.615	2.755	0.094	101.037	0.456 ~ 22381.560
Ratio of LVTH	0.045	0.025	−8.303	3.678	0.024	0.00025	1.83E-07 ~ 0.335

### 3.4 Construction and validation of the combined model

**Figure 4:**
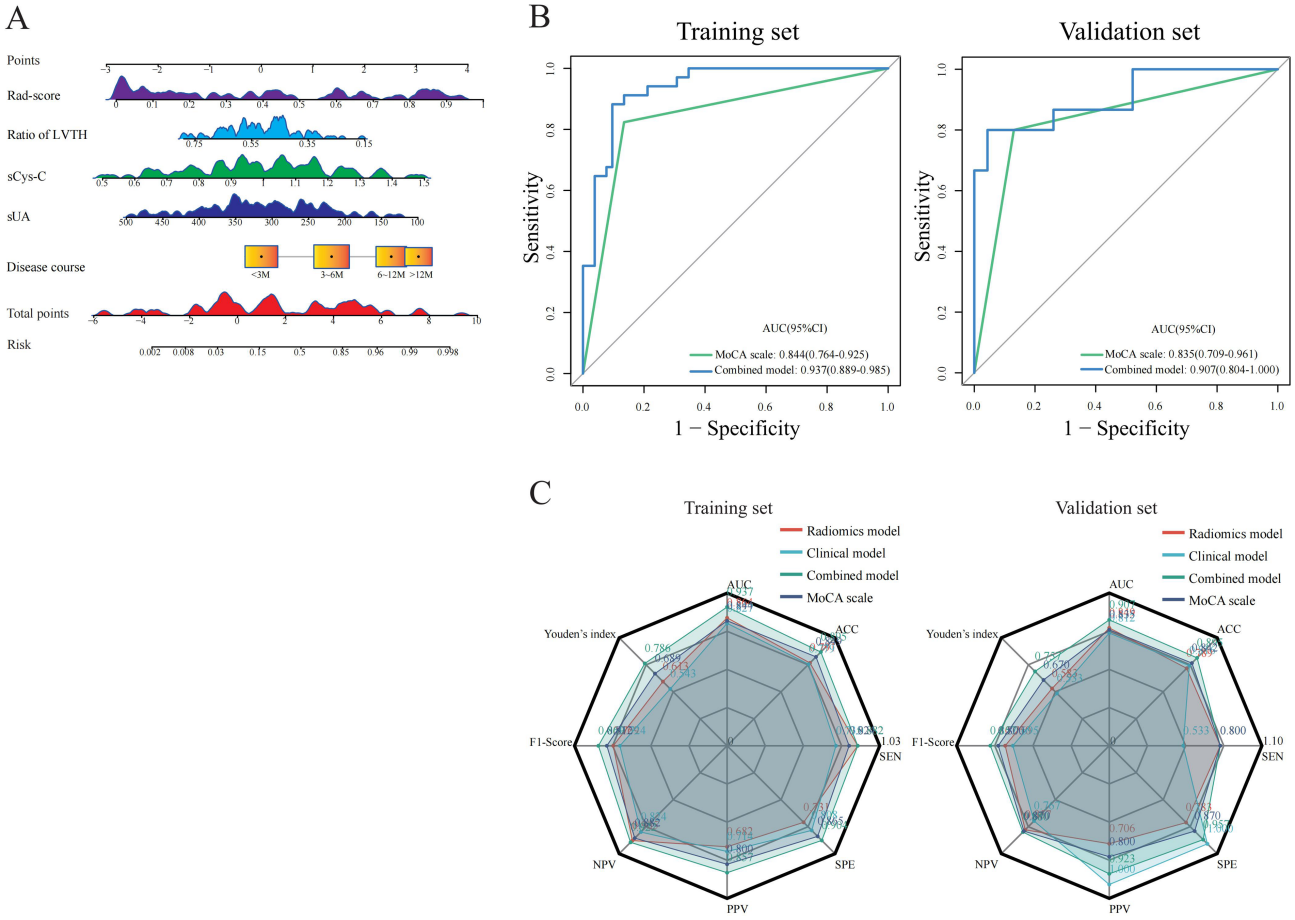
Visualization and comparison of constructed models with MoCA. **(A)** A nomogram plot for visualization of combined model in training set. **(B)** Performance comparisons of the combined model with MoCA. **(C)** Radar plots for comparisons of predictive characteristics among different models and MoCA.

Based on five independent predictors of MCI in patients with hydrocephalus, including Rad-score, disease duration, sUA, sCysC, and LVTA ratio, we developed a logistic regression algorithm to construct a combined model. Furthermore, we plotted a nomogram (Figure 4A). The model was developed using a training set. Subsequently, the predictive performance of the model was validated in a separate the validation set. The AUC for the combined model in the training and validation sets were 0.937(0.889 ~ 0.985) and 0.907(0.804 ~ 1.000), respectively (Figure 3B). All these indicated that the combined model exhibited the best discrimination. In both sets, the calibration curves closely aligned with the ideal curves, suggesting that the model fit was unbiased (Figure 3C). The model’s Brier scores in the training and validation sets were 0.093/0.112, the calibration slopes were 1.000/0.793, and the calibration intercepts were 0.000/−0.165, respectively, (Supplementary Table 2). The DCA demonstrated that the combined predictive model provided excellent net benefits across threshold probabilities ranging from 0.01 to 1.00 in the training set and from 0.05 to 0.95 in the validation set (Figure 3D).

### 3.5 Comparison of performance among the models

We compared the predictive performance of all established models on the training and validation sets, and the results indicated that all models exhibited good discriminative ability. The combined model achieved the optimal predictive performance. The DeLong test revealed that the AUC differences between the combined and clinical models were statistically significant in the training and validation sets (*Z* = 2.563 and 1.962, *p* = 0.010 and 0.048, respectively). The AUC difference between the combined and radiomics models was statistically significant in the training set (*Z* = 2.519, *p* = 0.012) but not in the validation set (*Z* = 0.828, *p* = 0.408). The AUC differences between the radiomics and clinical models were not statistically significant in both sets (*Z* = 0.582 and 0.386, *p* = 0.560 and 0.700, respectively). Calibration curves showed that the predicted results of the three models were consistent with the actual results. The results of the Hosmer–Lemeshow test demonstrated that the clinical model had good calibration accuracy in the training set (*χ*^2^ = 7.769, *p* = 0.456), and the radiomics and combined models had satisfactory calibration accuracies in both the training and validation sets (radiomics model *χ*^2^ = 8.953 and 5.216, *p* = 0.346 and 0.734; combined model *χ*^2^ = 5.995 and 7.618, *p* = 0.648 and 0.472, respectively), suggesting that there were no biases in fitting. The DCA revealed that the net benefit of all models was higher than the reference line, with a relatively large threshold interval. Among the models, the DCA curves of the combined model were consistently above those of the radiomics and clinical models, demonstrating that it had the best clinical benefit.

Furthermore, the MoCA scale is widely utilized in current clinical practice as a simple qualitative instrument for evaluating MCI. The AUC values of the MoCA scale were 0.844 (0.764 ~ 0.925) and 0.835 (0.709 ~ 0.961) in the training and validation sets, respectively. The combined model demonstrated higher AUC values than the MoCA scale in both sets (Figure 4B). The DeLong test revealed a significant difference in the training set (*Z* = 2.011, *p* < 0.05), with a consistent trend in the validation set that did not reach statistical significance (*Z* = 0.804, *p* > 0.05). In summary, the combined model has higher discriminative ability, calibration, and clinical net benefit than other models (Figure 4C; Supplementary Tables 1, 2).

### 3.6 Performance of resampling validation

Internal validation consistently showed robust performance across all predictive models, with the combined model displaying the greatest potential for generalizability.

During 5-fold cross-validation, the combined model demonstrated superior and stable predictive performance, achieving a mean AUC of 0.916 (±0.030). This was significantly higher than the AUCs of the clinical model (0.815 ± 0.030) and the radiomics model (0.853 ± 0.020). The standard deviations for all model performance metrics remained low, indicating that the models’ performance was minimally influenced by the randomness of data splitting, thus possessing good stability (Supplementary Figures 1A–C).

By bootstrap resampling validation with 500 iterations, model performance was corrected by calculating the optimism statistic. The AUC of the combined model was adjusted from 0.937 on the original training set to 0.910, indicating a small optimism bias. This suggests that while the initial performance estimate was slightly optimistic, the model’s performance remains strong. The corrected AUCs for the clinical and radiomics models were 0.809 (original: 0.827) and 0.851 (original: 0.864), respectively, also indicating good robustness (Supplementary Figures 1D–F). Notably, these bootstrap-corrected performance metrics closely aligned with the performance observed on our held-out internal validation set (30% of the total cohort), which was completely withheld from the model training process (Supplementary Tables 1, 3). This agreement further corroborates, from an internal data perspective, that all models possess excellent generalization ability.

### 3.7 Performance of temporal external validation

The generalizability of the models was conclusively confirmed on an independent temporal external validation cohort (*n* = 33).

Regarding discrimination, the AUCs (95% CI) for the combined model, radiomics model, and clinical model were 0.902 (0.794–1.000), 0.846 (0.716–0.975), and 0.808 (0.651–0.966), respectively. The combined model demonstrated the best discriminatory ability. However, DeLong’s test revealed no statistically significant differences in AUC between any pair of models (clinical vs. combined: *Z* = −1.125, *p* = 0.261; radiomics vs. combined: *Z* = −0.988, *p* = 0.323; clinical vs. radiomics: *Z* = −0.330, *p* = 0.741; Supplementary Table 1; Supplementary Figure 1G).

In terms of calibration, the calibration curves indicated good agreement between predicted probabilities and observed outcomes for all models, with the curve of the combined model being the closest to the ideal diagonal. The Hosmer-Lemeshow test suggested no significant lack of fit for any model (clinical model: *χ*^2^ = 15.419, *p* = 0.052; radiomics model: *χ*^2^ = 10.427, *p* = 0.236; combined model: *χ*^2^ = 12.086, *p* = 0.147; Supplementary Figure 1H). Detailed metrics, including the Brier score, calibration slope, and intercept, are provided in Supplementary Table 2.

In terms of clinical utility, the DCA revealed that across a broad spectrum of threshold probabilities, the net benefits of all three models surpassed those of the extreme strategy reference lines. The decision curve for the combined model consistently lay above those of the other models, offering a net clinical benefit over the broadest range of threshold probabilities (approximately 0.04 to 0.97). This indicates that employing the combined model for decision-making in this temporal external validation cohort results the optimal net benefit (Supplementary Figure 1I).

## 4 Discussion

This study established a radiomic model based on hippocampal T1WI-based radiomics model and a combined model incoporating both radiomic and clinical features. The results indicated that these models had satisfactory diagnostic performance in predicting the risk of MCI in patients with hydrocephalus. Compared to the MoCA scale, these models offered the advantages of objectivity and clinical applicability. Additionally, the combined model’s nomogram presented visual results, offering a new tool for accurate early diagnosis.

The clinical factor analysis revealed that a long duration of disease, low sUA, high sCys-C, and small LVTA ratio were independent risk factors for MCI. The results were consistent with those of previous reports (Lin et al., 2025; Lopez-Soley et al., 2021; Ren et al., 2024; Yan et al., 2022). However, there were some differences between our results and those of previous studies. Some studies found that education level was an independent predictor of cognitive impairment in older individuals (Guo et al., 2025; Yuan et al., 2024) which might contradict our findings. The diverse inclusion criteria and scope of previous studies may have led to different distribution of educational attainment among the participants. In our study, the distribution of educational attainment among the participants was relatively even. Moreover, the grading criteria for educational attainment were inconsistent across studies, which may have introduced misclassification bias, affecting the accuracy of effect estimates and consequently leading to different study outcomes. Furthermore, although populations across various geographical regions may have comparable years of education, they may have potential disparities in educational quality. These factors may influence the results. Additionally, some studies have shown that molecules such as amyloid *β*, tau protein, and *α*-synuclein could be associated with MCI (Hsu et al., 2024; McKenna et al., 2025; Ryczek et al., 2025). However, some of these proteins are not routinely tested in clinical practice. Our study showed that the total CSF protein correlated with MCI in univariate analysis, but the correlation was not statistically significant in the logistic regression model. We inferred that there was collinearity between total CSF protein levels and other clinical factors.

Neuroimaging serves as a crucial indicator and predictor of cognitive impairment. Numerous studies (Feng et al., 2019; Lin et al., 2025; Sung et al., 2024; Yang C. et al., 2024; Zhou et al., 2022) have shown that MCI is closely correlated with hippocampal injury. T1WI imaging scans capture microstructural changes in brain tissue. Consequently, we utilized radiomics technology to comprehensively characterize the T1WI features of the hippocampus and applied advanced algorithms to identify four key features associated with MCI, thereby enhancing the accuracy and stability of the model. Four radiomic features were found to be associated with MCI. Among these, wavelet. LLL-firstorder-Skewness evaluated the pixel density within the VOI, irrespective of spatial information among voxels. It reflected the differences in the distribution of internal gray intensity, suggesting that the occurrence of MCI tends to be heterogeneous and uneven (Mannina et al., 2024). In our study, MCI patients exhibited lower hippocampal skewness, indicating a more negatively skewed intensity distribution, which may potentially reflect a reduction in certain tissue components or signal homogenization. We hypothesized that this alteration may reveal macroscopic changes in tissue composition within the hippocampus. The GLSZM, which reflects the spatial distribution of gray levels and more complex inter-pixel dependencies in the hippocampus, indicated that patients with MCI exhibited more greater regional heterogeneity and a more complex spatial distribution of gray levels compared to others (Bicci et al., 2024). We observed that this feature was diminished in MCI patients, suggesting increased regional inhomogeneity and a more intricate gray-level spatial distribution or texture within the hippocampal structure of MCI patients. Additionally, the GLRLM revealed significant differences in the roughness, complexity, and homogeneity of hippocampal textures and was associated with atrophy and arrangement disorders of hippocampal cells in patients with MCI (Zhang et al., 2019). We inferred that alterations in this feature might reflect early damage to white matter microstructures that is challenging to detect with conventional imaging. Such damage could directly impede efficient neural information transmission, affect neural circuit efficiency, and thus contribute to the progression of MCI. The NGTDM assesses gray-level differences and spatial interrelationships between each pixel and its neighboring pixels, characterizes the dynamic range of intensities locally, and more accurately quantifies the inhomogeneity within the VOI (Gourtsoyianni et al., 2017). We hypothesize that this feature correlates with the microscopic contrast between different tissue components within the hippocampus. In summary, our Rad-score is not an abstract mathematical construct but incorporate pathophysiological information from various dimensions pertinent to cognitive decline in the context of hydrocephalus. These dimensions include tissue composition (Skewness), macroscopic structure (LargeAreaEmphasis), microstructure/fiber architecture (GrayLevelNonUniformity), and local textural contrast (Strength). It represents a comprehensive imaging biomarker that reflects the “hippocampal health status” or a quantitative “hippocampal health index.” A higher Rad-score indicates more severe cumulative damage to the hippocampus within the pathological environment of hydrocephalus, suggesting a diminished capacity to maintain normal cognitive function and a correspondingly increased risk of progressing to MCI.

Utilizing these four optimal features, we constructed the radiomics model using a machine learning logistic regression algorithm. Machine learning concentrates on the cross-validation and iterative enhancement of algorithms and emphasizing the predictive performance and generalization capabilities of models mathematically. This method can uncover the intrinsic relationships between variables, and its complexity greatly surpasses that of traditional models (Fuse et al., 2023).

To further enhance the diagnostic performance of the radiomics model, we integrated clinical and MRI features to construct a combined predictive model. The results indicated that this combined model exhibited optimal predictive performance in predicting MCI for patients with hydrocephalus. Regarding the goodness-of-fit for the combined model, the calibration curve suggested that the predicted probabilities were consistent with the actual probabilities. However, in the validation set, when the predicted probabilities were within the 25–55% range, the calibration curve of the model shifted toward the lower right, suggesting that the model might overestimate the risk of MCI positivity in this interval. This potential overestimation should be carefully considered in clinical practice. The significant fluctuations observed in the DCA of the combined model within the high-threshold range (0.8–1.0) in the training set can be attributed to the small-sample effect at high thresholds and the inherent mathematical sensitivity of the net benefit calculation formula. This is a common statistical phenomenon and does not indicate an intrinsic flaw in our model. It is important to note the potential instability of results within this high-threshold interval. Therefore, in clinical practice, the focus should be directed toward the medium- and low-threshold intervals (0.2–0.6), which offer greater clinical utility for informing decision-making. Furthermore, bootstrap resampling validation with 500 iterations demonstrated that the DCA remained highly stable within the clinically relevant threshold range (0.2–0.6), whereas it exhibited marked variability in the 0.8–1.0 threshold interval. This contrast robustly confirms that fluctuations in the high-threshold range originate from statistical instability rather than systematic model bias, thereby reinforcing the reliability of the model within the primary range for clinical decision-making (Supplementary Figure 2). However, the combined predictive model innovatively integrated radiomics features and clinical factors to comprehensively characterize lesions, breaking through the limitations of a single method and improving its predictive accuracy. Furthermore, we developed a nomogram to visualize the model (Xue et al., 2022), transforming the complex predictive model into an intuitive and easily understandable graphical interface. It greatly simplifies the model interpretation process and enables clinicians to quickly and accurately assess the risk status of patients (Han et al., 2024; Yang X. et al., 2024). As a valuable auxiliary tool, nomograms enhance the accuracy of disease diagnosis (Yu et al., 2023).

This study established a comprehensive and rigorous validation framework, progressing from multifaceted internal validation to prospective temporal external validation, which significantly strengthens the reliability and scientific rigor of our findings. The internal validation results indicated that our model performance estimates were robust, with statistical correction effectively mitigating the risk of over-optimism. The successful temporal external validation elevates the demonstration of model robustness to a higher level. It confirms that our models maintain excellent predictive performance when applied to new patients from the future, who may reflect subtle shifts in clinical workflows or population distributions. The combined model’s AUC of 0.902 on the independent temporal validation set, which aligns remarkably well with its bootstrap-corrected internal performance (AUC: 0.910), constitutes the cornerstone of our evidence for its powerful generalizability. This result substantially reduces the possibility that model performance was overestimated due to a single random data split or over-reliance on data patterns from a specific time period, strongly suggesting its potential for translation into a clinical tool. Although DeLong’s test indicated that the differences between models did not reach statistical significance, the consistent numerical superiority of the combined model across discrimination, calibration, and clinical utility (DCA) supports its potential as a superior predictive tool. This is likely attributable to the effective integration of complementary information from radiomic features and clinical factors.

The sample size in this study is justified based on the following: (1) EPV criterion: The effective events per variable (EPV) for the combined model was 6.8, which conforms to contemporary research indicating that an EPV > 5, when combined with appropriate statistical techniques such as penalized regression and resampling validation, significantly mitigates the risk of overfitting. (2) Multifaceted validation: Through rigorous feature engineering, multiple dimensionality reduction procedures, and extensive internal as well as temporal external validation, the stability and generalizability of the model were thoroughly ensured and confirmed. Thus, the sample size employed in this study is sufficient to support the development of a stable and reliable prediction model.

Some limitations of our study must be acknowledgement. First, the models developed herein are based on a complete dataset, which may restrict their applicability to more diverse populations that include individuals with missing data. Sceond, this is a retrospective study that utilized T1WI sequences (with a slice thickness of 6 mm and anisotropic voxels) configured for routine clinical scanning rather than being optimized for hippocampal radiomics research. The use of thick slices and anisotropic acquisition may introduce partial volume effects, potentially affecting the robustness of the radiomic features extracted. To minimize this issue, we implemented several measures in our analytical pipeline, such as rigorous image quality control with manual segmentation, image resampling to a uniform spatial resolution, and selection of highly stable features based on the ICC. Nonetheless, we recognize that the non-ideal imaging protocol represents an inherent limitation of this study. Future prospective studies should use standardized 3D high-resolution isotropic sequences (e.g., MPRAGE) to further validate and improve the performance and reproducibility of our model. Third, during the model construction process, this study did not employ batch effect correction methods such as ComBat, which we acknowledge as an important factor to consider in future validation on multi-center data and as a key area for improvement in subsequent research. Fourth, this study utilized a single-center design with a limited sample source. To mitigate the risk of model overfitting, we implemented rigorous feature engineering along with systematic multiple dimensionality reduction strategies to identify the most predictive features for model development. The model underwent stringent internal validation and was further evaluated through prospective temporal external validation, which collectively demonstrated its robustness and generalizability across multiple levels, thereby enhancing the reliability of the study findings. However, as all data were derived from a single center, the clinical applicability of the model requires further validation using external datasets from multi-center, multi-device, and heterogeneous patient populations in the future to ultimately establish its universal applicability. Fifth, the T1WI imaging scan represents only a single radiomics modality, and we only explored the influence of hippocampal global radiomics features on model performance. Therefore, exploring smaller hippocampal subregional features in early MCI and incorporating multi-modal imaging data in the radiomics analysis may improve the predictive accuracy of the models. Additionally, In this study, while manual segmentation helps ensure segmentation accuracy, it is time-consuming and operator-dependent, which may limit the model’s broad adoption in clinical practice. Therefore, we plan to focus future efforts on developing and validating a deep learning-based, automated, and robust tool for hippocampal segmentation. Our ultimate goal is to establish a comprehensive, end-to-end automated clinical workflow that integrates raw image input with output of MCI risk prediction.

In summary, we developed models using clinical data, hippocampal T1WI radiomic data, and a combination of these datasets to predict early MCI in patients with secondary hydrocephalus. The findings indicated that the integrated model, which combined Rad-scores with clinical features, exhibited superior predictive capabilities and achieved optimal performance. The nomogram of this combined model is more objective, quantifiable, and highly reproducible compared to the MoCA scale. Consequently, it offers an effective, personalized, visual, and non-invasive tool for clinical diagnosis and decision-making.

## Data Availability

The original contributions presented in the study are included in the article/Supplementary material, further inquiries can be directed to the corresponding authors.

## Footnotes

1
http://www.R-project.org

2
http://www.python.org

3
http://www.itksnap.org

4
http://www.rtog.org

5
https://pyradiomics.readthedocs.io
