# Supplementary material for: Hippocampal T1WI radiomics- and clinical feature-based models for predicting early mild cognitive impairment in secondary hydrocephalus
Source: Front Aging Neurosci. 2025 Dec 16;17:1672254. doi: 10.3389/fnagi.2025.1672254 (PMC12748200; doi:10.3389/fnagi.2025.1672254)
Supplement: Supplementary file 1 [file Data_Sheet_1.docx]

Supplementary Table 1. Performance of different models in prediction of MCI

| Dataset | Model | AUC | 95% CI | Accuracy | Sensitivity | Specificity | Positive Predictive Value | Negative Predictive Value | F1 Score | Youden’s index |
| --- | --- | --- | --- | --- | --- | --- | --- | --- | --- | --- |
| Train set | Clinical | 0.827 | 0.736~0.919 | 0.779 | 0.735 | 0.808 | 0.714 | 0.824 | 0.724 | 0.543 |
|  | Radiomics | 0.864 | 0.790~0.937 | 0.791 | 0.882 | 0.731 | 0.682 | 0.905 | 0.769 | 0.613 |
|  | Combined | 0.937 | 0.889~0.985 | 0.895 | 0.882 | 0.904 | 0.857 | 0.922 | 0.869 | 0.786 |
|  | MoCA | 0.844 | 0.764~0.925 | 0.849 | 0.824 | 0.865 | 0.800 | 0.882 | 0.812 | 0.689 |
| Test set | Clinical | 0.812 | 0.666~0.957 | 0.737 | 0.800 | 0.727 | 0.632 | 0.842 | 0.706 | 0.527 |
|  | Radiomics | 0.849 | 0.724~0.974 | 0.790 | 0.733 | 0.826 | 0.733 | 0.826 | 0.733 | 0.559 |
|  | Combined | 0.907 | 0.804~1.000 | 0.816 | 0.800 | 0.826 | 0.750 | 0.864 | 0.774 | 0.626 |
|  | MoCA | 0.835 | 0.709~0.961 | 0.842 | 0.800 | 0.870 | 0.800 | 0.870 | 0.800 | 0.670 |
| Temporal external validation set | Clinical | 0.808 | 0.651~0.966 | 0.758 | 0.786 | 0.737 | 0.688 | 0.824 | 0.734 | 0.523 |
|  | Radiomics | 0.846 | 0.716~0.975 | 0.727 | 0.826 | 0.794 | 0.647 | 0.813 | 0.726 | 0.620 |
|  | Combined | 0.902 | 0.794~1.000 | 0.818 | 0.824 | 0.841 | 0.833 | 0.810 | 0.828 | 0.665 |

Supplementary Table 2. Calibration Performance of different Models

| Dataset | Model | Brier Score | Calibration  Slope | Calibration  Intercept | χ²  (H-L test) | p*  (H-L test) |
| --- | --- | --- | --- | --- | --- | --- |
| Train set | Clinical | 0.160 | 1.000 | 0.000 | 7.769 | 0.456 |
|  | Radiomics | 0.157 | 1.000 | 0.000 | 8.953 | 0.346 |
|  | Combined | 0.093 | 1.000 | 0.000 | 5.995 | 0.648 |
| Test set | Clinical | 0.180 | 0.854 | -0.657 | 15.959 | 0.043 |
|  | Radiomics | 0.164 | 1.290 | 0.742 | 5.216 | 0.734 |
|  | Combined | 0.112 | 0.793 | -0.165 | 7.618 | 0.472 |
| Temporal external validation set | Clinical | 0.175 | 0.940 | 0.028 | 15.419 | 0.052 |
|  | Radiomics | 0.170 | 0.943 | 0.170 | 10.427 | 0.236 |
|  | Combined | 0.135 | 0.791 | 0.331 | 12.086 | 0.147 |

Supplementary Table 3. Comparison of AUCs among different models across various validation strategies

| Model | Training set  (apparent) | 70/30 Split Validation set | 5-fold cross-validation  (mean ± SD) | Bootstrap-corrected  (500 repetitions) | Temporal external validation set |
| --- | --- | --- | --- | --- | --- |
| Clinical | 0.827 (0.736~0.919) | 0.812 (0.666~0.957) | 0.815 ± 0.030 | 0.809 (0.715~0.901) | 0.808 (0.651~0.966) |
| Radiomics | 0.864 (0.790~0.937) | 0.849 (0.724~0.974) | 0.853 ± 0.020 | 0.851 (0.781~0.932) | 0.846 (0.716~0.975) |
| Combined | 0.937 (0.889~0.985) | 0.907 (0.804~1.000) | 0.916 ± 0.030 | 0.910 (0.856~0.961) | 0.902 (0.794~1.000) |
