# Supplementary figures and images for: Hippocampal T1WI radiomics- and clinical feature-based models for predicting early mild cognitive impairment in secondary hydrocephalus
Source: Front Aging Neurosci. 2025 Dec 16;17:1672254. doi: 10.3389/fnagi.2025.1672254 (PMC12748200; doi:10.3389/fnagi.2025.1672254)

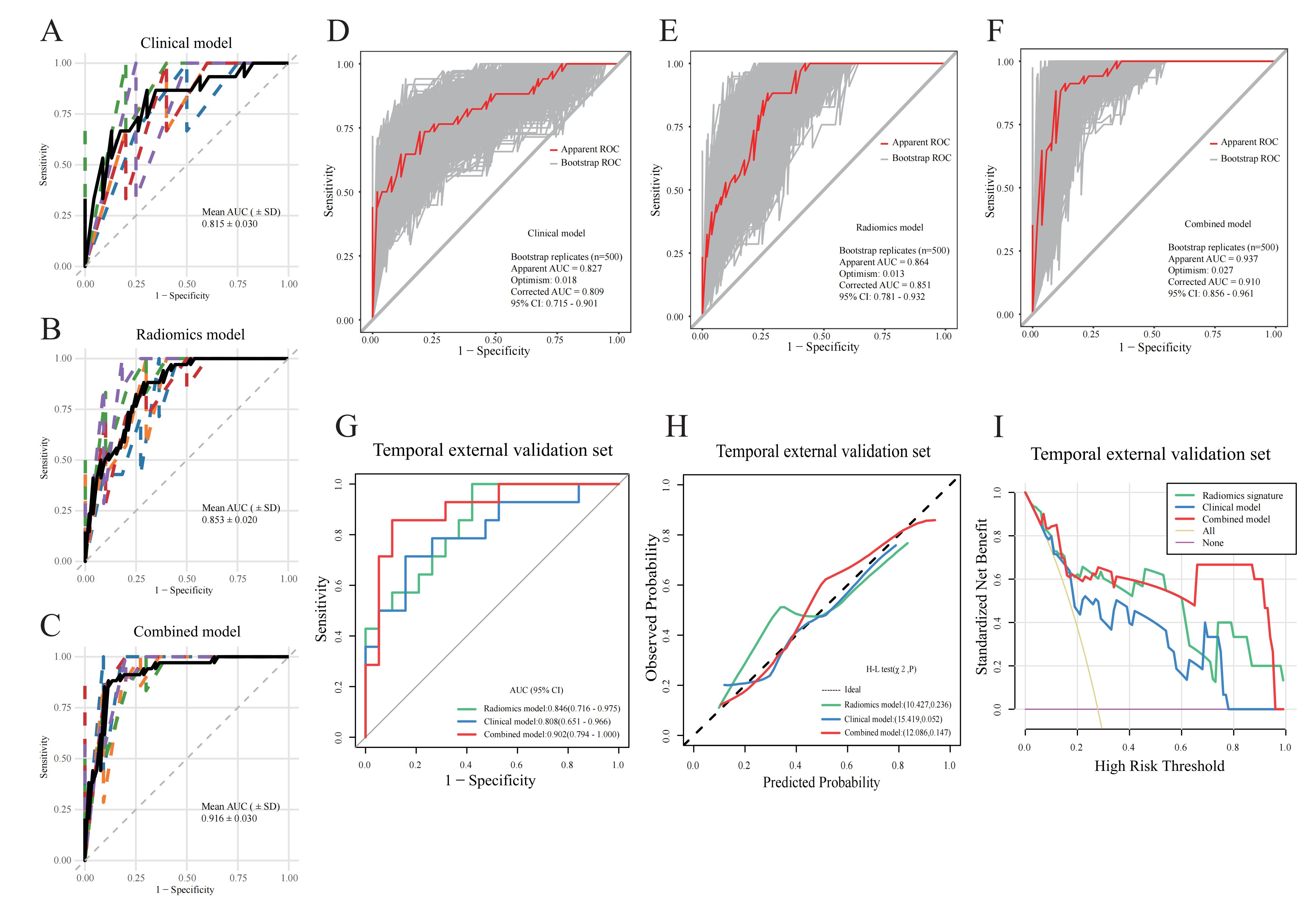

Supplement: Supplementary file 2 [file Image_1.jpeg]

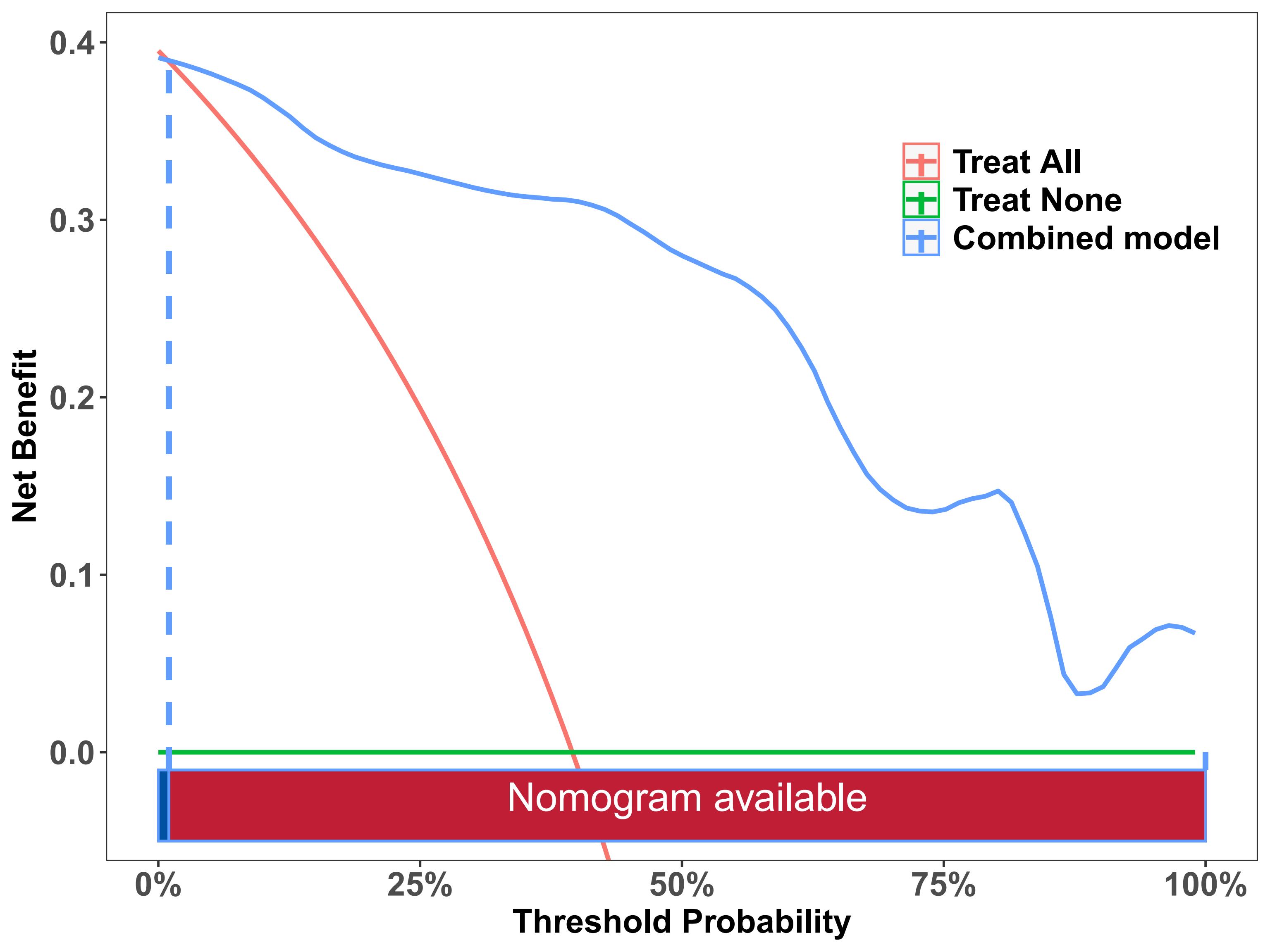

Supplement: Supplementary file 3 [file Image_2.jpeg]
